# Dual Carbonic Anhydrase IX/XII Inhibitors and Carbon Monoxide Releasing Molecules Modulate LPS-Mediated Inflammation in Mouse Macrophages

**DOI:** 10.3390/antiox10010056

**Published:** 2021-01-05

**Authors:** Emanuela Berrino, Simone Carradori, Andrea Angeli, Fabrizio Carta, Claudiu T. Supuran, Paolo Guglielmi, Cecilia Coletti, Roberto Paciotti, Helmut Schweikl, Francesca Maestrelli, Elisabetta Cerbai, Marialucia Gallorini

**Affiliations:** 1 Neurofarba Department, University of Florence, Via U. Schiff 6, 50019 Florence, Italy; emanuela.berrino@unifi.it (E.B.); andrea.angeli@unifi.it (A.A.); fabrizio.carta@unifi.it (F.C.); claudiu.supuran@unifi.it (C.T.S.); elisabetta.cerbai@unifi.it (E.C.); 2 Department of Pharmacy, “G. d’Annunzio” University of Chieti-Pescara, via dei Vestini 31, 66100 Chieti, Italy; cecilia.coletti@unich.it (C.C.); r.paciotti@unich.it (R.P.); marialucia.gallorini@unich.it (M.G.); 3 Centre of Advanced Research in Bionanoconjugates and Biopolymers Department, “Petru Poni” Institute of Macromolecular Chemistry, 700487 Iasi, Romania; 4 Department of Drug Chemistry and Technologies, Sapienza University of Rome, P.le A. Moro 5, 00185 Rome, Italy; paolo.guglielmi@uniroma1.it; 5 Department of Conservative Dentistry and Periodontology, University Hospital Regensburg, University of Regensburg, D-93042 Regensburg, Germany; helmut.schweikl@ukr.de; 6 Department of Chemistry, University of Florence, Via U. Schiff 6, 50019 Florence, Italy; francesca.maestrelli@unifi.it

**Keywords:** CO-RMs, carbonic anhydrase inhibitor, cobalt, macrophages, inflammation, carbon monoxide

## Abstract

Low concentrations of carbon monoxide (CO) were reported to exhibit anti-inflammatory effects when administered in cells by suitable chemotypes such as CO releasing molecules (CO-RMs). In addition, the pH-modulating abilities of specific carbonic anhydrase isoforms played a crucial role in different models of inflammation and neuropathic pain. Herein, we report a series of chemical hybrids consisting of a Carbonic Anhydrase (CA) inhibitor linked to a CO-RM tail (CAI/CO-RMs). All compounds and their precursors were first tested in vitro for their inhibition activity against the human CA I, II, IX, and XII isoforms as well their CO releasing properties, aiming at corroborating the data by means of molecular modelling techniques. Then, their impact on metabolic activity modulation of RAW 264.7 mouse macrophages for 24 and 48 h was assessed with or without lipopolysaccharide (LPS) stimulation. The compounds were shown to counteract the inflammatory stimulus as also indicated by the reduced tumor necrosis factor alpha (TNF-α) release after treatment. All the biological results were compared to those of *N*-acetylcysteine (NAC) as a reference antioxidant compound. Within the series, two CAI/CO-RM hybrids (**1** and **2**), bearing both the well-known scaffold able to inhibit CAs (acesulfame) and the cobalt-based CO releasing portion, induced a higher anti-inflammatory effect up to 48 h at concentrations lower than NAC.

## 1. Introduction

The idea to make use of Carbon Monoxide (CO) for the management of human pathologies by means of its controlled administration is convincingly sustained by an increasing number of scientific evidence [1]. To date cardiovascular and inflammatory-based diseases, either in vitro and in vivo, are the most advanced and reliable models which account for the therapeutic use of CO [2,3]. Safety of inhaled CO has been evaluated in a completed clinical trial on healthy volunteers, showing that doses up to 250 ppm are well tolerated with only minimal side effects observed [4]. Examples on the therapeutic significance of CO include the promising results for the treatment of lung injuries [5], as well as a selection of clinical trials currently ongoing [6,7]. The effects of hyper-inflammation in inflammatory diseases are largely attributed to the impairment of several signaling pathways associated with cell responses toward oxidative stress, including the Nrf2/inducible heme oxygenase-1 (HO-1)/CO molecular axis [8,9]. Under oxidative stress, the HO-1 enzyme degrades heme group, leading to the production of potent antioxidant, anti-inflammatory, and bactericidal mediators (biliverdin, bilirubin, and CO) and monocytes/macrophages rely on abundant induction of the HO-1/CO pathway [10]. The overexpression of HO-1 during altered intracellular redox state confirms its role as an important component of stress response and suggests the cytoprotective effects exerted by CO, acting as gasotransmitter along with NO and H_2_S [11]. Administration of external sources of CO by means of gas inhalation or through CO releasing molecules (CO-RMs) [12], aims at simulating the biological effects of the HO-1/CO molecular pathway. However, reaching the precise CO concentrations produced endogenously might be very difficult and remains an open challenge.

First reported in 2002, CO-RMs turned out to be very promising therapeutic agents, with many derivatives reported so far evaluated in several disease models [13,14]. CO-RMs chemical structure is usually characterized by the presence of a transition metal core able to coordinate CO residues, which in turn is linked to an organic portion easy to manipulate. Among the most investigated CO-RMs reported so far in models of inflammation, the Ru-based CORM-2 and -3 showed to suppress inflammatory phenotype mainly acting on oxidative stress and NO production, two of the main initiators of the inflammatory cascade [2]. In an in vitro model of lipopolysaccharide (LPS)-stimulated macrophages, CORM-2 and -3 reduced pro-inflammatory cytokine/growth factor (e.g., tumor necrosis factor alpha (TNF-α)) production and reduced the activation of polymorphonuclear cells in rat endothelial cells incubated with CORM-3 [15,16,17,18]. Many intracellular targets of CO have been identified so far. However, significant differences in the biological effects and intracellular mechanisms are observed when CO is administered by inhalation or through CO-RM species [19]. Such differences are mainly ascribed to diverse CO release rates and tissue concentrations upon administration. Although the metallo-organic portion in CO-RMs ensures a better control of CO release over the time, it affects any prediction reliability on either biological property and safety profile, which are far easier when the inhalation route is applied. This is particularly true for the less investigated CO-RM family based on the dicobalt(0) hexacarbonyl (DCH) complex [20,21,22]. Straightforward synthesis of CO-RM/DHCs allows to largely modulate the organic portion linked to the CO-RM section, (i.e., named the “drug sphere”). The nature of the drug spheres showed to deeply influence the biological properties of the CO-RM itself. For instance, slight modifications on the organic scaffold of the acetylsalicylic acid-DCH complex, which is reported to possess strong antiproliferative effect on various cell lines, showed to modulate the biological activity [21]. The CO release rate from DCH complexes is also reported to be lower when compared to Ru or Mn containing CO-RMs, and often not detectable in cell assays making use of fluorescent probes [13,22,23]. Intriguingly, CO release from DCHs was also found to occur after metal oxidation, as reported in a work describing the synthesis of fumaric acid ester DCH derivatives and their in vitro evaluation in murine RAW 264.7 macrophages [24]. This work reported that the CO release was stimulated by the oxidative environment and that the observed anti-inflammatory properties were related to the modulation of the cell oxidative state. These interesting features of DCH/CO-RMs recently led some of us to synthesize dual-acting Carbonic Anhydrase (CA)/CO-RM hybrids for the management of chronic inflammation in a rat model of rheumatoid arthritis, where the chemical species produced because of the oxidative stress processes typical of this disease would have triggered the CO release from the complex. Indeed, proof-of-concept studies have revealed that the concurrent inhibition of the specific CA isoform aberrant activity importantly reduced pain symptoms in in vivo rheumatoid arthritis models, being involved in the pathogenesis and maintenance of inflammatory-related diseases and in the dysregulation of cellular immunity processes [25]. The promising preliminary results fostered our interest in investigating such relatively poorly explored CO-RM class, although possessing very interesting properties.

In the present work, we synthesized new DCH/CO-RMs, endowed with different organic portions linked to the CO-RM sphere, aiming at exploring how the organic ligand could modulate their CO releasing profiles as well as their potential antioxidant and anti-inflammatory properties in murine cell line model, under LPS-induced oxidative stress conditions.

## 2. Experimental Procedures

### 2.1. Chemistry

Solvents and reagents were used as supplied without further purification (Sigma-Aldrich, Milan Italy). Where mixtures of solvents are specified, the stated ratios are volume:volume. Stuart^®^ melting point apparatus SMP1 has been used to measure the melting points (uncorrected). ^1^H and ^13^C NMR spectra were recorded at 400 and 101 MHz, respectively, on a Bruker spectrometer using CDCl_3_ and DMSO-*d*_6_ as the solvents at room temperature. Chemical shifts are expressed as *δ* units (parts per millions) relative to the solvent signal. Coupling constants *J* are valued in Hertz (Hz). Purification on column chromatography was carried out using silica gel (high purity grade, pore size 60 Å, 230–400 mesh particle size). All the operations were monitored by TLC performed on 0.2 mm thick silica gel-aluminium backed plates (60 F254, Merck). Visualization was carried out under ultra-violet irradiation (254 nm). Where given, systematic compound names are those generated by ChemBioDraw Ultra 12.0 following IUPAC conventions. Microanalyses were performed with a Perkin-Elmer 260 elemental analyzer (PerkinElmer, Inc., Waltham, MA, USA) for C, H, and N and the results were within ±0.4% of the theoretical values.

The analyses for purity determination were done by using the Shimadzu Prominence-*i* LC-2030C 3D system endowed with autosampler, binary pump, column oven and photodiode array detector (PDA) [26]. The separation was accomplished by using a biphenyl column (Kinetex 5 µm Biphenyl, 100 Å, Phenomenex, Bologna, Italy) as stationary phase. A binary mobile phase gradient, obtained by mixing water (solvent A) and acetonitrile (solvent B) at a constant flow of 1.00 mL min^−1^, was employed for the elution. The eluent composition was 50% of solvent B, at the first, kept constant for 3 min then raised up to 100% in 9 min and maintained at this value for other 3 min. Finally, the percentage was taken back to the starting value of 50% of solvent B for the column reconditioning (5 min), to a total run of 20 min (the last 5 min were not acquired). Except for compounds **1**, **4**, **8,** and **10**, endowed with lower solubility and getting solutions of about 0.5 mg mL^−1^, all the analytes were dissolved in acetonitrile at the concentration of about 1 mg mL^−1^. Three microliters were directly injected for the HPLC analysis after sample preparation and the acquisitions accomplished at the wavelength of 211 nm. All the compounds exhibited HPLC purity > 95%. The solvents used in the HPLC analysis were acetonitrile, purchased from Carlo Erba Reagents and mQ water 18 MΩ cm, obtained from Millipore’s Direct-Q3 system.

### 2.2. Syntheses of the Propargylated Compounds ***a**–**l***

The precursor, endowed with the corresponding OH/SH/NH moiety, (1 equiv.) in anhydrous DMF (10 mL) was stirred with anhydrous K_2_CO_3_ (1.3 equiv.) at room temperature for 1 h. Propargyl (prop-2-ynyl) bromide (1.5 equiv) was added slowly to the reaction mixture and stirring was continued for 23 h at room temperature. The reaction was diluted with water (50 mL) and extracted with CHCl_3_ (3 × 20 mL), dried (anhydrous Na_2_SO_4_), and the solvent removed. The crude product obtained was purified by column chromatography on silica gel with the eluent being a mixture of *n*-hexane and ethyl acetate to afford the title compounds [27].

### 2.3. Characterization Data of the Novel Propargylated Compounds

6-methyl-3-(prop-2-yn-1-yl)-1,2,3-oxathiazin-4(3*H*)-one 2,2-dioxide (**b**): colorless sticky liquid, 56% yield. ^1^H NMR (400 MHz, CDCl_3_): *δ* 2.23 (s, 3H, CH_3_), 2.36–2.38 (m, 1H, ≡CH), 4.56–4.58 (m, 2H, NCH_2_), 5.85 (bs, 1H, =CH). ^13^C NMR (101 MHz, CDCl_3_): *δ* 19.8 (CH_3_), 31.5 (NCH_2_), 73.5 (C≡), 75.9 (HC≡), 104.3 (=CH), 159.3 (=C-O), 162.3 (NC=O). Anal. Calcd for C_7_H_7_NO_4_S: C, 41.79; H, 3.51; N, 6.96. Found: C, 41.98; H, 3.53; N, 6.92.

3-benzoyl-8-(prop-2-yn-1-yloxy)-2*H*-chromen-2-one (**f**): light brown solid, mp 165–167 °C, 76% yield. ^1^H NMR (400 MHz, CDCl_3_): *δ* 2.56 (s, 1H, ≡CH), 4.90 (s, 2H, OCH_2_), 7.24–8.06 (m, 9H, Ar). ^13^C NMR (101 MHz, CDCl_3_): *δ* 57.1 (OCH_2_), 76.6 (C≡), 77.5 (HC≡), 117.9 (Ar), 119.0 (Ar), 121.7 (Ar), 124.7 (Ar), 127.3 (Ar), 128.6 (Ar), 129.6 (Ar), 133.9 (Ar), 136.1 (Ar), 144.8 (Ar), 145.5 (Ar), 157.7 (Ar), 191.6 (OC=O). Anal. Calcd for C_19_H_12_O_4_: C, 74.99; H, 3.98. Found: C, 74.70; H, 4.01.

3-benzoyl-6-(prop-2-yn-1-yloxy)-2*H*-chromen-2-one (**g**): brown solid, mp 160–162 °C, 86% yield. ^1^H NMR (400 MHz, CDCl_3_): *δ* 2.56 (s, 1H, ≡CH), 4.75 (s, 2H, OCH_2_), 7.12–8.04 (m, 9H, Ar). ^13^C NMR (101 MHz, DMSO-*d*_6_): *δ* 56.6 (OCH_2_), 79.2 (C≡), 79.3 (HC≡), 113.8 (Ar), 118.0 (Ar), 119.1 (Ar), 122.2 (Ar), 127.3 (Ar), 129.2 (Ar), 130.0 (Ar), 134.4 (Ar), 136.5 (Ar), 145.4 (Ar), 149.5 (Ar), 154.1 (Ar), 158.6 (Ar), 192.2 (OC=O). Anal. Calcd for C_19_H_12_O_4_: C, 74.99; H, 3.98. Found: C, 74.83; H, 3.96.

ethyl 2-oxo-8-(prop-2-yn-1-yloxy)-2*H*-chromene-3-carboxylate (**h**): white solid, mp 127–129 °C, 58% yield. ^1^H NMR (400 MHz, CDCl_3_) *δ* 1.35–1.39 (t, *J* = 7.2 Hz, 3H, CH_3_), 2.60 (s, 1H, ≡CH), 4.34–4.38 (q, *J* = 7.2 Hz, 2H, OCH_2_), 4.77 (s, 2H, OCH_2_), 6.92–6.94 (d, *J* = 8.1 Hz, 2H, ArH), 7.50–7.52 (d, *J* = 8.0 Hz, 1H, ArH), 8.49 (s, 1H, ArH). Anal. Calcd for C_15_H_12_O_5_: C, 66.17; H, 4.44. Found: C, 66.09; H, 4.47.

1-(5-(2-methoxyphenyl)-3-(4-(prop-2-yn-1-yloxy)phenyl)-4,5-dihydro-1*H*-pyrazol-1-yl)ethan-1-one (**i**): white solid, mp 109–110 °C, 54% yield. ^1^H NMR (300 MHz, CDCl_3_): *δ* 2.46 (s, 3H, CH_3_), 2.53 (bs, 1H, ≡CH), 2.96–3.03 (dd, *J* = 3.9 Hz, 17.6 Hz, 1H, H-pyr), 3.63–3.73 (dd, *J =* 11.7 Hz, 17.7 Hz, 1H, H-pyr), 3.84 (s, 3H, OCH_3_), 4.71 (bs, 2H, OCH_2_), 5.08–5.86 (dd, *J =* 3.9 Hz, 11.7 Hz, 1H, H-pyr), 6.85–6.89 (m, 2H, ArH), 6.97–7.01 (m, 3H, ArH), 7.19–7.24 (m, 1H, ArH), 7.66–7.69 (d, *J* = 8.1 Hz, 2H, ArH). ^13^C NMR (75 MHz, DMSO-*d*_6_): *δ* 21.9 (CH_3_), 41.6 (OCH_3_), 55.4 (CH_2_), 55.7 (CH), 58.8 (OCH_2_), 76.0 (C≡), 78.0 (HC≡), 110.8 (Ar), 115.0 (Ar), 120.6 (Ar), 125.1 (Ar), 125.6 (Ar), 128.1 (Ar), 128.6 (Ar), 129.0 (Ar), 154.7 (Ar), 156.0 (Ar), 159.1 (Ar), 168.7 (NC=O). Anal. Calcd for C_21_H_20_N_2_O_3_: C, 72.40; H, 5.79; N, 8.04. Found: C, 72.30; H, 5.82; N, 8.09.

1-phenyl-2-(4-(prop-2-yn-1-yloxy)phenyl)-1*H*-benzo[*d*]imidazole (**l**): brown amorphous powder, 34% yield. ^1^H NMR (300 MHz, CDCl_3_): *δ* 2.50 (bs, 1H, ≡CH), 4.64 (bs, 2H, OCH_2_), 6.85–6.90 (m, 2H, ArH), 7.19–7.34 (m, 5H, ArH), 7.44–7.53 (m, 5H, ArH), 7.85–7.88 (m, 1H, ArH). ^13^C NMR (75 MHz, DMSO-*d*_6_): *δ* 55.7 (OCH_2_), 76.0 (C≡), 78.1 (HC≡), 110.4 (Ar), 114.6 (Ar), 119.5 (Ar), 122.9 (Ar), 123.0 (Ar), 123.2 (Ar), 127.4 (Ar), 128.6 (Ar), 129.9 (Ar), 130.9 (Ar), 136.9 (Ar), 137.1 (Ar), 142.6 (Ar), 152.0 (C=N), 158.5 (Ar). Anal. Calcd for C_22_H_16_N_2_O: C, 81.46; H, 4.97; N, 8.64. Found: C, 81.29; H, 5.00; N, 8.60.

### 2.4. Synthesis of DCH/CO-RMs ***1**–**10***

The appropriate alkyne derivative (1.0 equiv) was dissolved in tetrahydrofuran (THF) (15 mL) and then dicobalt octacarbonyl (1.1 equiv) was added. The dark-red mixture was stirred at room temperature for 1 h (TLC monitoring). Then, the solvent was removed under vacuum to give a black-red solid residue which was purified by silica gel column chromatography eluting with the appropriate mixture of EtOAc/*n*-hexane to afford the titled compounds [25].

### 2.5. Characterization Data of DCH/CO-RMs ***1**–**10***

6-methyl-4-(prop-2-yn-1-yloxy)-1,2,3-oxathiazine 2,2-dioxide hexacarbonyldicobalt (**1**): red powder, 67% yield. ^1^H NMR (400 MHz, DMSO-*d*_6_): *δ* 2.27 (s, 3H, CH_3_), 5.21 (s, 2H, OCH_2_), 6.36 (s, 1H, ≡CH), 6.72 (s, 1H, =CH). ^13^C NMR (101 MHz, DMSO-*d*_6_): *δ* 19.4, 45.4, 75.1, 104.1, 159.8, 163.7, 199.6.

6-methyl-3-(prop-2-yn-1-yl)-1,2,3-oxathiazin-4(3*H*)-one 2,2-dioxide hexacarbonyldicobalt (**2**): red powder, 57% yield. ^1^H NMR (400 MHz, DMSO-*d*_6_): *δ* 2.27 (s, 3H, CH_3_), 5.21 (s, 2H, NCH_2_), 6.36 (s, 1H, ≡CH), 6.72 (s, 1H, =CH). ^13^C NMR (101 MHz, DMSO-*d*_6_): *δ* 19.4, 45.8, 75.1, 104.1, 160.2, 163.7, 199.6.

1-isopropyl-4-methyl-2-(prop-2-yn-1-yloxy)benzene hexacarbonyldicobalt (**3**): red powder, 80% yield. ^1^H NMR (400 MHz, DMSO-*d*_6_): *δ* 1.13 (bs, 6H, 2 x CH_3_), 2.27 (bs, 3H, CH_3_), 5.26 (s, 2H, OCH_2_), 6.72 (s, 1H, ≡CH), 6.86 (s, 1H, ArH), 6.89 (s, 1H, ArH), 7.10 (s, 1H, ArH). CH signal is overlapped with DMSO signal.

1-bromo-4-(prop-2-yn-1-yloxy)benzene hexacarbonyldicobalt (**4**): red powder, 72% yield. ^1^H NMR (400 MHz, DMSO-*d*_6_): *δ* 5.31 (s, 2H, OCH_2_), 6.81 (s, 1H, ≡CH), 6.96–6.98 (d, *J* = 8.0 Hz, 2H, ArH), 7.48–7.50 (d, *J* = 8.0 Hz, 2H, ArH).

methyl 2-(prop-2-yn-1-ylthio)benzoate hexacarbonyldicobalt (**5**): red powder, 64% yield. ^1^H NMR (400 MHz, DMSO-*d*_6_): *δ* 3.79 (bs, 3H, OCH_3_), 4.57 (bs, 2H, SCH_2_), 6.58 (bs, 1H, ≡CH), 7.29 (bs, 1H, ArH), 7.60 (bs, 2H, ArH), 7.89 (bs, 1H, ArH).

3-benzoyl-8-(prop-2-yn-1-yloxy)-2*H*-chromen-2-one hexacarbonyldicobalt (**6**): red powder, 60% yield. ^1^H NMR (400 MHz, DMSO-*d*_6_): *δ* 5.56 (s, 2H, OCH_2_), 6.83 (s, 1H, ≡CH), 7.37 (s, 1H, ArH), 7.44 (s, 1H, ArH), 7.56 (s, 2H, ArH), 7.71 (s, 1H, ArH), 7.91 (s, 2H, ArH), 8.32 (s, 1H, ArH), 8.41 (s, 1H, ArH).

3-benzoyl-6-(prop-2-yn-1-yloxy)-2*H*-chromen-2-one hexacarbonyldicobalt (**7**): red powder, 56% yield. ^1^H NMR (400 MHz, DMSO-*d*_6_): *δ* 5.39 (s, 2H, OCH_2_), 6.84 (s, 1H, ≡CH), 7.49–7.51 (d, *J* = 8.8 Hz, 1H, ArH), 7.56–7.58 (m, 4H, ArH), 7.71 (s, 1H, ArH), 7.92–7.94 (d, *J* = 6.8 Hz, 2H, ArH), 8.36 (s, 1H, ArH).

ethyl 2-oxo-8-(prop-2-yn-1-yloxy)-2*H*-chromene-3-carboxylate hexacarbonyldicobalt (**8**): red powder, 71% yield. ^1^H NMR (400 MHz, DMSO-*d*_6_): *δ* 1.29–1.32 (t, *J* = 7.0 Hz, 3H, CH_3_), 4.26–4.31 (q, *J* = 14.0 Hz, 2H, CH_2_), 5.51 (s, 2H, OCH_2_), 6.86 (s, 1H, ≡CH), 7.00–7.03 (d, *J* = 8.8 Hz, 1H, ArH), 7.12 (s, 1H, ArH), 7.88–7.90 (d, *J* = 8.8 Hz, 1H, ArH), 8.74 (s, 1H, ArH).

1-(5-(2-methoxyphenyl)-3-(4-(prop-2-yn-1-yloxy)phenyl)-4,5-dihydro-1*H*-pyrazol-1-yl)ethan-1-one hexacarbonyldicobalt (**9**): red solid, 44% yield. ^1^H NMR (400 MHz, DMSO-*d*_6_): *δ* 2.32 (s, 3H, CH_3_), 2.92–2.96 (bd, *J* = 16 Hz, 1H, H-pyr), 3.82 (bs, 4H, H-pyr + OCH_3_), 5.37 (bs, 2H, OCH_2_), 5.65 (bs, 1H, H-pyr), 6.80 (bs, 1H, ≡CH), 6.88 (bs, 1H, ArH), 7.05 (bs, 3H, ArH), 7.24 (bs, 1H, ArH), 7.73 (bs, 2H, ArH), 8.31 (bs, 1H, ArH).

1-phenyl-2-(4-(prop-2-yn-1-yloxy)phenyl)-1*H*-benzo[*d*]imidazole hexacarbonyldicobalt (**10**): red solid, 66% yield. ^1^H NMR (400 MHz, DMSO-*d*_6_): *δ* 5.34 (bs, 2H, OCH_2_), 6.99 (bs, 1H, ≡CH), 7.03–8.20 (m, 12H, ArH), 8.32 (bs, 1H, ArH).

### 2.6. CA-Inhibitory Activity Evaluation

An Applied Photophysics stopped-flow instrument has been used for assaying the CA catalysed CO_2_ hydration activity. Phenol red (at a concentration of 0.2 mM) has been used as indicator, working at the absorbance maximum of 557 nm, with 20 mM Hepes (pH 7.5) as buffer, and 20 mM Na_2_SO_4_ (for maintaining constant the ionic strength), following the initial rates of the CA-catalyzed CO_2_ hydration reaction for a period of 10–100 s. The CO_2_ concentrations ranged from 1.7 to 17 mM for the determination of the kinetic parameters and inhibition constants. For each inhibitor at least six traces of the initial 5–10% of the reaction have been used for determining the initial velocity. The uncatalyzed rates were determined in the same manner and subtracted from the total observed rates. Stock solutions of inhibitor (0.1 mM) were prepared in distilled-deionized water and dilutions up to 0.01 nM were done thereafter with the assay buffer. Inhibitor and enzyme solutions were preincubated together for 15 min at room temperature prior to assay, to allow for the formation of the E-I complex. The inhibition constants were obtained by non-linear least-squares methods using PRISM 3 and the Cheng–Prusoff equation, as reported earlier, and represent the mean from at least three different determinations. All CA isoforms were recombinant ones obtained in-house as reported earlier [28,29].

### 2.7. CO-Release Assay

Gaseous CO was purchased from Rivoira (Milan, Italy); all the other reagents were of analytical grade and obtained from Sigma (Milan, Italy). UV-Vis absorption spectra were recorded using a Shimadzu UV-1900 UV-Vis Spectrophotometer from 275 to 700 nm at the scanning rate of 200 nm/min in a disposable plastic cuvette (path length 0.44 cm). Second derivative spectra were obtained using the Lab Calc program (Galactic Industries, Salem, NH). For the differentiation process, the Savitzky–Golay method was applied using 25 data points. No changes in the wavelength or in the bandwidth were observed when the number of points was increased or decreased. A stock solution of lyophilized horse heart Mb was freshly prepared by dissolving the protein in phosphate buffered saline flushed with N_2_ (PBS, 0.01 M, pH 7.4) to a 20 μM final concentration. Then, 2 mL of this solution were put in a cuvette and the UV-Vis absorption spectrum of met-Mb was recorded. Then, the solution was split in half: in the first half (reference) 10 μL of sodium dithionite (30 mg/mL) were added, and the UV-Vis spectrum of deoxy-Mb was recorded. Then, the solution was flushed with CO gas and the Mb-CO spectrum was acquired. The second half (sample) was reduced with sodium dithionite and, after recording a spectrum, a CO-RM DMSO solution was added and gently mixed, to a final CO-RM concentration of 20 μM. The solution was overlaid with 300 μL of light mineral oil to prevent Mb oxygenation and CO escaping and the absorption spectrum at t = 0 was recorded. Then, the sample was kept at 37 °C and spectra were recorded every 30 min for 5 h. During the assay, further additions of freshly prepared sodium dithionite solution were made when necessary. After 300 min the sample was flushed with CO gas to determine the total Mb concentration at the end of the assay. The assay was repeated three times for each tested compound and the mean of the three results for each time point was calculated.

### 2.8. Computational Details

The first step in our computational approach is the construction of a reliable model of the complexes formed between hCA (isoform IX and XII) and ligands **1** and **2** in order to evaluate their mechanism of action, their structure activity relationship (SAR) and the corresponding energetics. As a matter of fact, to the best of our knowledge, no X-ray structures of the hCA/**a** and hCA/**b**, nor hCA/DCH-ligands complexes are available. Because of this, we used a computational protocol based on molecular docking and geometry optimization calculations. The structures of the hCA IX-mimic and XII isoforms were prepared by means of Protein Preparation Wizard [30], obtained from 6UGZ [31] and 1JD0 [32] pdb files, respectively.

A molecular docking study of ligands **a** and **b** was carried out by employing the SP approach implemented in Glide [30], adopting the OPLS_2005 force field [33,34]. Because for the Co atom and DCH moiety no parameters are available which allow a direct use of a docking procedure, the best ranking poses of **a** e **b** were used to manually build the corresponding DCH derivatives, **1** and **2**, respectively. In order to reduce the computational burden of the subsequent geometry optimizations, we generated reduced ligand-receptor complexes, selecting only the residues around 10 Å from the ligand, and capping the incomplete residues by adding H atoms. The following reduced ligand-receptor complexes hCA IX/**a**, hCA IX/**b**, hCA IX/**1**, hCA IX/**2**, hCA XII/**a**, hCA XII/**b**, hCA XII/**1**, hCA XII/**2**, and the free reduced receptors, hCA IX and hCA XII, were optimized in water using density functional tight-binding (DFTB) theory as implemented in XTB software [35,36], adopting the GFN2-xTB method [37]. The coordinates of the backbone atoms were kept frozen in the optimization calculations. For each ligand, **a**, **b**, **1,** and **2**, a conformational search was preliminary performed by using the CREST script [38] and the lowest energy conformer structures were then minimized by using XTB. The GB/SA method implemented in the XTB software was employed to simulate the effects of water solvation.

The electronic energies of the ligand-receptor complexes, free receptors and ligands were used to compute the theoretical electronic binding energy in solution, ∆E_calc_, according to

∆E_calc_ = E(ligand-receptor) − [E(receptor) + E(ligand)] (1)

Then, to assess the reliability of the predicted binding poses, we performed a correlation study between the ∆E_calc_ values and the experimental binding free energies, ∆G_exp_, obtained by experimental *K*_I_ applying the following Equation (2):

∆G_exp_ = −RTln(1/*K*_I_) (2)

where R is the gas constant, 8.314 JK^−1^mol^−1^, and T equals to 298.15 K. All the structures were manipulated by using Maestro [26], which was also used to produce Figures 6 and 7.

### 2.9. Cell Culture

RAW264.7 mouse macrophages (ATCC TIB71) were cultivated in RPMI 1640 medium (PAN Biotech, Aidenbach, Germany) supplemented with L-glutamine, sodium-pyruvate, 2.0 g/L NaHCO_3_, 10% fetal bovine serum (FBS), and 1% penicillin/streptomycin (Life Technologies, Gibco BRL, Eggenstein, Germany) following standard culture techniques previously reported [39].

Macrophages (5 × 10^3^ cells/well) from routine culture were sub-cultivated on 96-well Clear Flat Bottom TC-treated Culture Microplate (Falcon^®^, Corning Inc., NY, USA) at 37 °C for 48 h. Next, cells were exposed to loading concentrations (0–200 μM) of compounds **1**–**10** for 24 and 48 h as shown in the Supplementary section. Cells were afterwards stimulated with 0.1 μg/mL LPS (Sigma, Taufkirchen, Germany) and exposed to increasing concentrations (0–400 μM) of compounds **1**–**10** and NAC (0–10 mM) (Sigma, Taufkirchen, Germany) for 24 and 48 h.

### 2.10. Cell Metabolic Activity Assay (MTT)

After the established time points, the medium was discarded and replaced with a fresh one containing 0.5 mg/mL MTT (3-[4,5-dimethylthiazol-2-yl]-2,5-diphenyl tetrazolium bromide) (Sigma-Aldrich, Milan, Italy). Cells were afterwards incubated for 1 h at 37 °C. After having discarded the MTT medium, a same volume of DMSO was added to each well and samples were incubated for 20 min at 37 °C and afterwards incubated at room temperature under gentle shaking for additional 10 min. The absorbance was measured at 540 nm using a spectrophotometer (Infinite F200, TECAN, Mainz, Germany) and optical density values were collected using Magellan software (version 6.2). The percentage of metabolically active cells and thus viable, was calculated setting the untreated control as 100%. Two independent experiments were performed under the same experimental conditions (n = 6) [40].

### 2.11. TNF-α Release

Amounts of tumor necrosis factor-α (TNF-α) in cell supernatants after 24 h were determined using a standard ELISA kit (BD Pharmingen, San Diego, CA, USA) following the manufacturer’s instructions. Briefly, microwells were coated with 100 μL/well of Capture Antibody diluted in the coating buffer, sealed, and incubated overnight at 4 °C. After having washed wells, plates were blocked with 200 µL/well Assay Diluent and incubated for 1 h at room temperature. Next, wells were washed and 100 µL of each standard or sample or control were pipetted into the appropriate wells and incubated for 2 h at room temperature. Then, wells were washed, 100 µL/well of Working Detector (Detection Antibody + Streptavidin-HRP reagent) were added and incubated for 1 h at room temperature. After having pipetted a same volume of Substrate Solution for 30 min and having afterwards added 50 µL/well of the Stop solution, the absorbance was read at 450 nm using a spectrophotometer (Infinite F200, TECAN, Mainz, Germany) and optical density values were collected using Magellan software (version 6.2). The lower detection limit for TNF-α was 15.6 pg/mL. Levels of cytokines detected in untreated cell cultures were below the detection limit, and thus set to the lowest detectable levels in standard curves. The TNF-α concentration was normalized on the MTT optical densities and expressed as the fold increase of TNF-α (pg/mL) released from cells stimulated with 0.1 μg/mL LPS alone for 24 h set as 1.

### 2.12. Statistics

Statistical analysis was established by the analysis of variance (one-way ANOVA) followed by Tukey’s post-hoc test (GraphPad Prism, GraphPad Software, San Diego, CA, USA, version 5.0). Results were expressed as means ± SD. Values of *p* < 0.05 were considered statistically significant.

## 3. Results and Discussion

### 3.1. Chemistry

**Figure 1:**
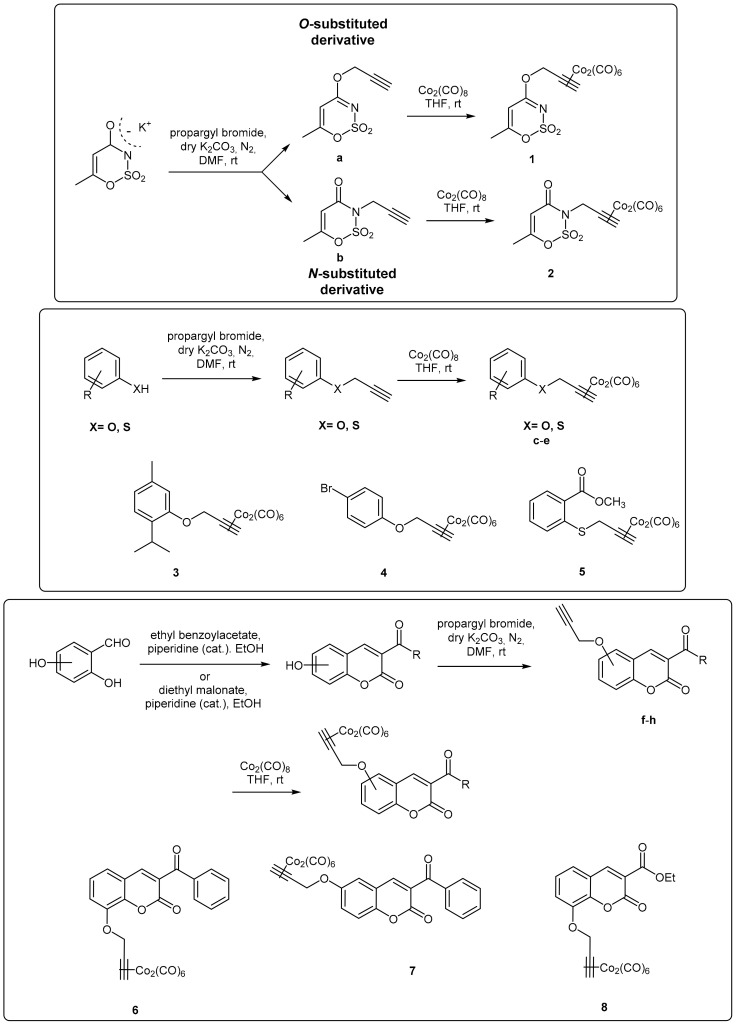
Acesulfame-, aryl-, and coumarin-based CAI/CO-RMs.

**Figure 2:**
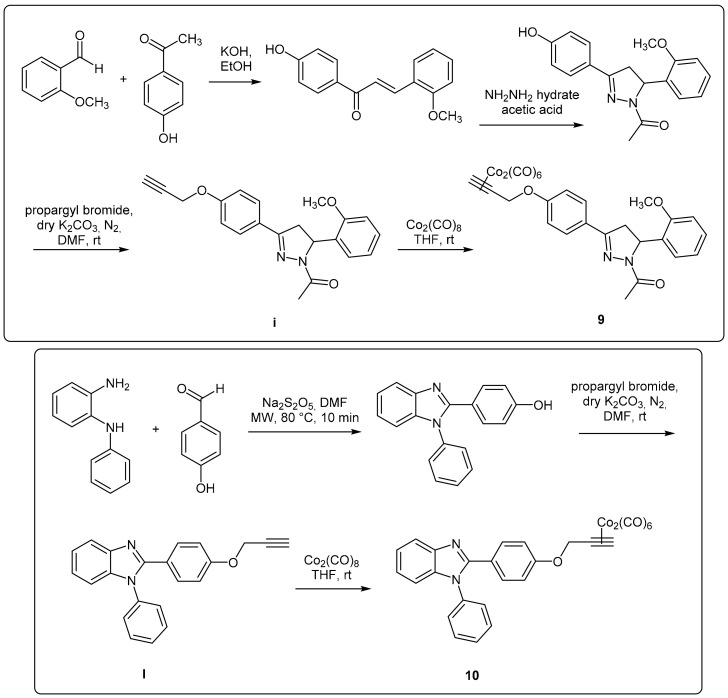
Pyrazoline- and benzimidazole-based CAI/CO-RMs.

In this work, DCH/CO-RMs presenting different “drug spheres” are reported. Ten compounds were synthesized according to the general procedure reported in Figure 1 and Figure 2 based on procedures published in our previous works [41,42,43,44].

After having purified the core nuclei endowed with OH/SH/NH moieties, a propargylation reaction in dry *N*,*N*’-dimethylformamide (DMF) and in the presence of anhydrous potassium carbonate gave the terminal alkynes suitable for the reaction with dicobalt(0) octacarbonyl in THF to afford the desired CO-RMs in good yield. Before submitting to the biological evaluations, all the synthesized compounds have been fully characterized by spectroscopic data and their purity evaluated through chromatographic analysis (HPLC). A gradient elution approach with a binary mobile phase composed by water and acetonitrile, was employed. All the analyzed compounds were >95% HPLC pure (see chromatograms in Figure S1). Low cost of the reagents, high yields and easy availability of the compounds prompted us to widely explore the biological targets of these molecules.

### 3.2. Carbonic Anhydrase Inhibition

Acesulfame (**1**, **2**) and coumarins (**6**–**8**) are chemical entities known to possess CA inhibitory properties [42,45,46]. DCH/CO-RM derivatives **1**–**10** were therefore investigated as inhibitors of the hCAs I, II, IX, and XII by means of the Stopped-Flow CO_2_ hydrase assay [32]. The inhibition data, compared to those of the standard sulfonamide inhibitor acetazolamide (**AAZ**), are reported in Table 1.

In agreement with the literature data, compounds **1** and **2**, *O-* and *N*-substituted acesulfame derivatives, respectively, and coumarins **6**–**8** showed to act as potent and selective CA inhibitors toward the tumor associated isoforms IX and XII with *K*_I_ spanning from medium nanomolar to micromolar range (56.3–8112 nM). Compounds **2** and **6**, in particular, selectively inhibited hCA XII over hCA I, II and IX, whereas compounds **1**, **7**, and **8** showed to inhibit both tumor-associated isoforms hCA IX, and XII. Of particular note is the *O*-substituted acesulfame derivative **1**, which proved to be the most potent with *K*_I_ values of 56 nM and 788.4 nM against hCA IX and XII, respectively. As expected, compounds **3**–**5**, **9**, and **10**, which do not possess any function able to inhibit CA, proved to be inactive against the selected isoforms (*K*_I_ > 10,000 nM).

Usually, the insertion of a DCH moiety in the propargyl tail is known to preserve the CA inhibitory properties of the compounds as well as their selectivity profile [25]. However, this general behavior slightly mismatched with the data observed for some of the propargylated precursors of compounds **1**–**10**, devoid of the CO releasing group (molecules **a**-**l**, Table S1). Indeed, the absence of the DCH complex on the drug sphere resulted in differences in the inhibitory activity and selectivity data. Compounds **f** and **g**, the DCH-free analogues of derivatives **6** and **7**, while maintained unchanged their inhibitory activity against hCA XII, exhibited better inhibitory activity against hCA IX (*K*_I_ **f** = 695.0 nM; *K*_I_ **g** = 315.5 nM), than the analogues bearing DCH group (*K*_I_ **6** > 10,000 nM; *K*_I_ **7** = 8112 nM).

Compounds **a** and **b** showed better inhibitory activity against isoform XII with respect to their counterpart endowed with CO releasing group (derivatives **1** and **2**, respectively). On the contrary, the effects elicited by DCH on the inhibitory activity against hCA IX were opposite among the two derivatives. The addition of DCH on compound **a** improved the affinity against this isoform (*K*_I_ = 252.0 nM) leading to compound **1** (*K*_I_ = 252.0 nM), the best inhibitor of this series; conversely, compound **b** exhibited a loss of inhibitory activity after the CO releasing group linkage. Interestingly, while compound **8** was completely ineffective against all the evaluated hCA isoforms, its DCH-free analogue **g**, exhibited a high nanomolar range inhibitory activity against the tumor related isoforms (*K*_I_ hCA IX = 315.5 nM; *K*_I_ hCA XII = 353.0 nM), accounting for an “interfering” effect elicited by the monoxide releasing moiety.

### 3.3. Carbon Monoxide Release Assay

**Figure 3:**
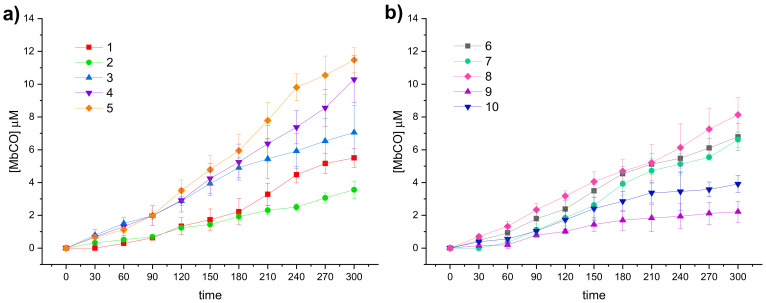
CO release profiles of DCH/CO-RMs **1**–**5** (**a**) and **6**–**10** (**b**), as obtained from the spectrophotometric assay (mean value of 3 results).

The CO releasing properties of the compounds reported in this study were evaluated by using the myoglobin (Mb)-based spectrophotometric assay, with modifications from the literature procedures [48]. Briefly, DCH/CO-RMs, dissolved in DMSO, were added to a 20 μM deoxy-Mb (II) solution to get a final CO-RM concentration of 20 μM and UV-Vis spectra were recorded every 30 min for 5 h. The absorption variations in the Soret region of the deoxy-Mb spectrum were analyzed over time, applying the second derivative approach to clearly distinguish each Mb form present in the mixture during the assay. The relative amount of Mb-CO formed in presence of deoxy-Mb at each time point was calculated by substituting the absorbances measured at Mb-CO and deoxy-Mb absorption maxima (422 and 431 nm, respectively) in the equation reported by Smulevich et al. [49]. Multiplying the obtained value by the initial Mb concentration and applying a corrective factor to cope with the observed Mb degradation induced by sodium dithionite (used as Mb reducing agent) allowed to correctly calculate the Mb-CO concentration at each time point, as previously reported [25]. In Figure 3 the CO release profiles of the analyzed compounds are reported as concentration of formed Mb-CO over time (up to 3 h).

In agreement with the few data available from the literature for this scarcely investigated CO-RM family so far, the reported DCH/CO-RMs showed to be slow CO releasers, when compared to Ru, Fe or Mn containing CO-RMs [13]. In addition to representing an advantage when designing CO-RMs for therapeutic purposes, such an extended CO release over time allows also to finely appreciate the impact of the so-called “drug-sphere” in influencing the CO releasing properties from the “CO-RM sphere” [50]. As matter of fact, strong differences in the release profiles can be observed within the series here reported, characterized by a high structural diversity (Figure 3a,b). This study aims to define preliminary but significant Structure–Release Relationships (SRR) for DCH/CO-RMs by including compounds bearing the DCH group linked to very heterogeneous chemical moieties with different steric and electronic properties.

**Figure 4:**
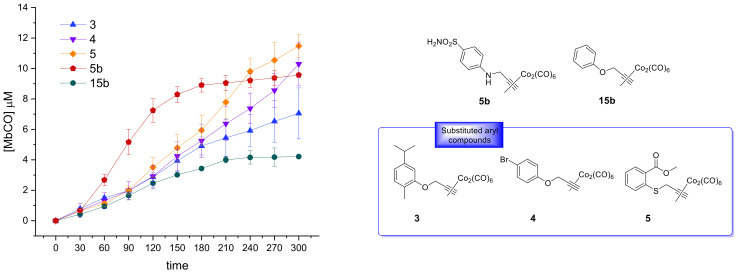
CO release profiles for the aryl substituted DCH/CO-RMs **3**–**5** and the reference compounds **5b** and **15b** (left), along with their chemical structures (right).

We also included in the SRR discussion compounds **5b** and **15b**, 4-SO_2_NH_2_ and 4-H aryl substituted DCH/CO-RMs respectively, previously reported by us as internal references (Figure 4) [25]. The release profiles of compounds **3**–**5**, belonging to the same chemical cluster of **5b** and **15b**, are also reported for comparison purposes (Figure 4).

**Figure 5:**
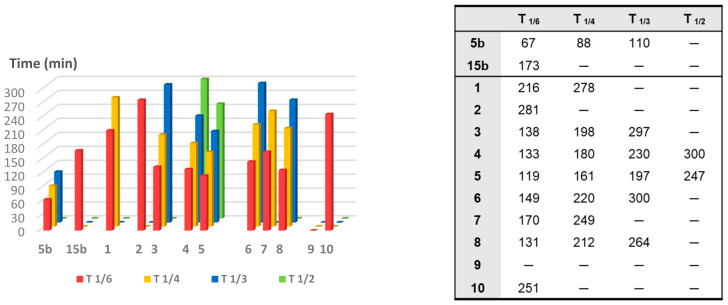
T_1/6_, T_1/4_, T_1/3_ and T_1/2_ values (defined as the time necessary for a CO-RM solution to produce a Mb-CO concentration equal, respectively, to 1/6, 1/4, 1/3, and 1/2 of its initial concentration) of the analyzed compounds based on the spectrophotometric assay. The bar graph (left) represents values reported in the table (right).

To compare the CO release rate from different CO-RMs, we calculated their T_1/X_ values, defined as the time necessary for a CO-RM solution to produce a Mb-CO concentration equal to 1/X (1/6, 1/4, 1/3, and 1/2) of its initial concentration, as reported in Figure 5.

With the only exception represented by dihydropyrazole-based compound **9**, the slowest and less efficient CO-RM within the series (2.2 μM of Mb-CO formed after 300 min), all the reported compounds can be compared by means of their T_1/6_ values. Aryl substituted compounds **3**–**5** and coumarin-based compounds **6**–**8** showed T_1/6_ values ranging between 119 and 170 min, whereas higher values were calculated for acesulfame derivatives **1** and **2** and for the benzimidazole-based compound **10** (216, 281, and 251 min, respectively). Interestingly, a superimposable CO release for the first 90 min can be noticed for the aryl substituted compounds **3**–**5**, with strong differences in their release profiles observed thereafter. This trend was not observed in the coumarin series **6**–**8**, where each compound showed different CO release efficiency only after 30 min of incubation and the gap between each compound remained quite constant even at longer incubation times. In particular, the coumarin-based compound **8**, bearing the CO-RM portion in position 8 and an ethyl ester substituent in position 3, showed to be the most efficient CO releaser within the coumarin series (T_1/3_ value of 264 min), followed by the 8-substituted coumarin **6**, bearing a phenone group in position 3 (T_1/3_ value of 300 min). The 6-substituted coumarin **7**, presenting the same phenone substituent in position 3, showed a similar CO release profile to **6**, but with higher T_1/6_ and T_1/4_ values. The data could suggest a favourable impact of the CO-RM insertion in position 8 of the coumarin ring, when compared to position 6, with a less crucial role played by the nature of the substituent in position 3. However, more studies are needed to confirm this hypothesis.

The slow CO release showed by the reported compounds allowed to calculate the T_1/2_ values for the aryl substituted compounds **4** and **5** only. Sulfur containing compound **5** showed a slow but intense CO release up to 240 min (9.8 μM of Mb-CO formed), furnishing 11.5 μM concentration of Mb-CO formed at 300 min. When compared to the reference compound **5b**, which provided a fast and sustained CO release for the first 120 min reaching the plateau after 180 min, it is clear how the aryl substitution pattern and the different heteroatom between the aryl and CO-RM section both play a crucial role in influencing the CO release rate. The presence of an electron withdrawing group (EWG), such as the Br atom or carbonyl and sulfonamide groups, as in compounds **4**, **5** and **5b**, respectively, seems to positively influence the CO release rate and efficiency from the DCH/CO-RMs. On the other hand, the insertion of electron donating groups (EDGs), such as the alkyl moieties present in compound **3**, or the absence of substituents, as for compound **15b**, showed to slow down the CO release.

For compounds **1** and **2**, originating from the two different acesulfame tautomers, the CO release rate was almost superimposable for the first 120 min. Thereafter, the *O*-alkylated compound **1** showed a more sustained CO release, when compared to the *N*-alkylated derivative **2**, providing the formation of 5.5 μM of Mb-CO after 300 min, more than 1/4 of the total Mb concentration. As for compound **2**, forming only 3.5 μM of Mb-CO at the same time point, a T_1/6_ value of 281 min could be calculated. The slow and controlled CO release from compounds **1** and **2** along with the hCA IX and XII inhibitory activity make them promising dual-acting therapeutic agents and useful tools to investigate the anti-inflammatory mode of action of DCH/CO-RMs.

### 3.4. Computational Characterization of hCA/DCH-Acesulfame Complexes

The experimental data of paragraph 2.2 indicate that the introduction of the DCH moiety in propargyl acesulfame binders affects their ability as hCA inhibitors and their selectivity against hCA isoforms. These findings are particularly interesting because one would expect the bulky DCH moiety to hamper the possibility of effective binding to hCA, thus resulting in a limited or decreased activity. However, this is the case only for some specific isoforms (e.g., hCA XII), others, on the contrary, show an enhanced activity upon DCH conjugation (like hCA IX). Moreover, CO release studies report that after 15 min, the preincubation time of hCA inhibition studies, the amount of released CO is almost negligible, suggesting that the ligand may interact with the receptor as a whole, supporting the hypothesis that the DCH moiety plays a role in the recognition process.

For this reason, it is important to try and understand the mechanism of interaction between the ligand and the receptor, focusing on the involvement of DCH moiety, which might be crucial to be able to modulate the ligand activity. To this aim we performed an explorative computational study of the binding mechanism and of the binding strength of some of the most active compounds presented in this work, namely the propargyl acesulfame **a** and **b**, and their DCH derivatives, **1** and **2**, to the hCA, isoforms IX-mimic and XII.

**Figure 6:**
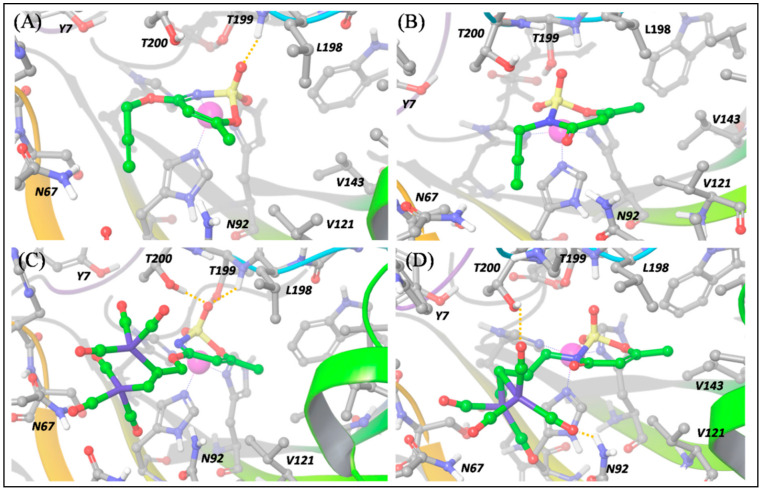
Predicted binding poses of **a** (panel (**A**)), **b** (panel (**B**)), **1** (panel (**C**)) and **2** (panel (**D**)) into hCA IX-mimic pocket. Hydrogen bond interactions are reported by orange dotted lines. The Zn^2+^ ion is represented as a purple sphere.

**Figure 7:**
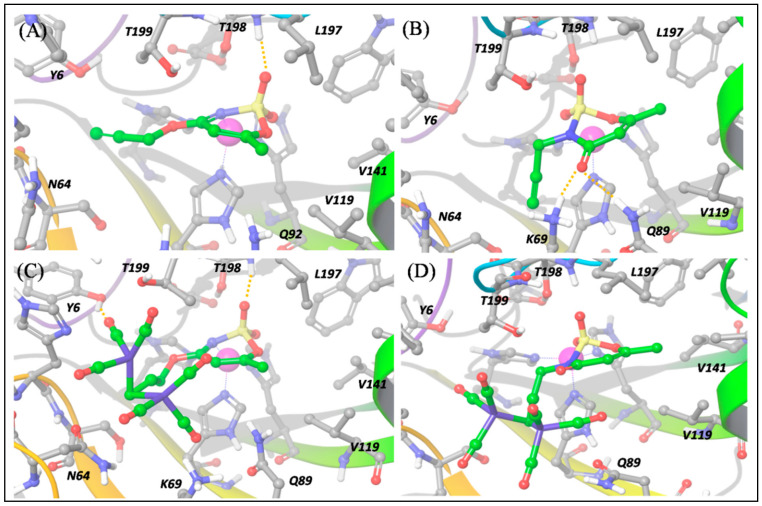
Predicted binding poses of **a** (panel (**A**)), **b** (panel (**B**)), **1** (panel (**C**)) and **2** (panel (**D**)) into hCA XII pocket. Hydrogen bond interactions are reported by orange dotted lines. The Zn^2+^ ion is represented as a purple sphere.

In particular, we carried out a molecular docking investigation, as described in the computational details section, which produced the binding modes reported in Figure 6; Figure 7 for the four compounds **a**, **1**, **b** and **2** against isoforms hCA IX-mimic and hCA XII, respectively.

For each binding mode, the electrostatic binding energies in solution were calculated by using the density functional tight-binding (DFTB) level of theory [35]. The binding energies were then correlated with the experimental free binding energies ∆G_exp_ (derived from the experimental *K*_I_ values). Indeed, the computational protocol employed here does not allow to directly compute free energies since an accurate estimation of thermal corrections and of the binding entropy is not possible. In any case, the entropic contribution is expected to be very similar within the set of calculations for each isoform, so that the computed electronic energies should qualitatively reflect the same trend as the free binding energies.

Table S2 reports the experimental ∆G_exp_ and the calculated ∆E_calc_ for the investigated compounds as well as the R^2^ values for the linear correlation for each isoform (also shown in Figures S2 and S3). A very good linear correlation was found with R^2^ values of 0.927 (onto hCA IX-mimic) and 0.924 (onto hCA XII), showing that our exploratory computational protocol might indeed be a reliable tool and that some important physical insights can be obtained from the visual observation of the binding poses which might shed some light onto the binding mechanism.

The predicted binding poses of **a** and **b** into hCA IX-mimic and XII binding pockets highlight the important role played by the *O*- and *N*-propargylation on acesulfame scaffold activity. As shown in Figure 6a, ligand **a**, the *O*-substituted derivative, interacts with the hCA IX binding pocket hitting the Zn^2+^ center by means of the N and O atoms; SO_2_ also establishes a hydrogen bond with the NH group of Thr199 (backbone). Moreover, the methyl group is involved in hydrophobic contacts with Val121, Val142 and Leu198. All these interactions are also established within hCA XII binding pocket (Figure 7a) and the binding pose is nearly the same.

On the contrary, for ligand **b**, the *N*-substituted binder, the binding pose cannot necessarily be the same in hCA IX and XII (Figure 6b and Figure 7b), because the N→Zn^2+^ interaction is hampered by the *N*-propargylation. For both isoforms, **b** is maintained in the pocket by the interaction of the acesulfame oxygen atoms with Zn^2+^. However, in hCA XII, **b** can form additional interactions by means of the carbonyl O atom in C5, free to interact with the positively charged Lys69 and with Gln89 (Figure 7b), which slightly stabilize the binding pose with respect to hCA IX, as reflected by computed ∆E and the experimental *K*_I_ values (Table S2).

Based on the predicted binding energies it can be concluded that the free N atom of acesulfame moiety is crucial to effectively interact with the metal center of hCA IX-mimic isoform and the *N*-propargylation, which disrupts this interaction, drastically reduces the binding affinity. On the contrary, the interaction with hCA XII is enhanced by the N-substitution, because the carbonyl group in C5 can now establish strong H bonds with the charged residue Lys69, increasing the ligand-receptor interaction.

The introduction of the DCH moiety, characterized by a huge steric volume, determines a distortion of the binding poses between the DCH acesulfame derivatives, **1** and **2**, and hCA IX. In details, the binding pose of **1**, compared to **a**, is characterized by the interaction between two O atoms and Zn^2+^, instead of N→Zn^2+^ interaction, and by hydrogen bonds between another O atom and Thr199 (NH) and the side chain of Thr200 (Figure 6c). In this pose, the DCH moiety can effectively avoid steric clashes, which leads to a stronger binding to hCA IX. The DCH conjugation also affects the interactions of the acesulfame moiety of ligand **2** with hCA IX-mimic, due to the steric clashes with nearby residues. The binding pose of *N*-substituted ligand **2** (Figure 6D) is thus predicted farther from the metal ion because of the DCH moiety lying close to the acesulfame scaffold and hampering the formation of H bonds with Thr200 and Thr199. As a result, only one O atom of SO_2_ group still maintains an interaction with Zn^2+^ while two CO molecules interact with Gln92 and Asn67. The binding efficiency is decreased with respect to its precursor, ligand **b** (Table S2).

When we consider the binding to the hCA XII isoform, besides a H bond between one CO group and Tyr6, the predicted binding pose of **1** maintains the N→Zn^2+^ interaction described for **a** (Figure 7C). This is possible because the hCA-XII isoform is characterized by the presence of the flexible residue, Lys69, instead of Gln67 (in hCA IX-mimic). This allows to fit the DCH moiety and to maintain the N→Zn^2+^ interaction. Nevertheless, because of the bulky DCH moiety, the interaction distance between N and Zn^2+^ is longer (2.26 Å) than in the precursor ligand **a** (2.05 Å) and the overall binding much less effective (Table S2).

The above-mentioned flexibility of hCA XII is however not sufficient to fit the DCH moiety of **2**, and only the O atom can still interact with Zn^2+^, as shown in Figure 7D. Moreover, the H bonds established by ligand **b** are disrupted and the binding becomes much less efficient than for its precursor **b**.

These results can thus contribute to rationalize some of the experimental outcomes, highlighting that the DCH moiety does play a role in hCA recognition. In particular, the interesting and rather unexpected selectivity of compound **1** against hCA IX isoform, can be explained by the strong H bonds established by the acesulfame moiety and by two O atoms coordinating the Zn^2+^ center, a consequence of the coordination to cobalt, which induces a rearrangement in the acesulfame scaffold binding mode.

### 3.5. Biological Evaluation

#### 3.5.1. Cell Metabolic Activity

To evaluate the effect on cell viability of compounds **1**–**10** in terms of metabolic activity modulation (MTT assay), loading concentrations (0–400 μM) of molecules were administered for 24 and 48 h to a mouse macrophage cell line, namely RAW 264.7 cells (Figure S4). This widely used murine cell line represents a suitable in vitro system for initially screening newly synthesized compounds, especially for anti-inflammatory purposes [51]. As a matter of comparison, increasing concentrations of the antioxidant *N*-acetyl cysteine (NAC) were tested in the same cell line. The effect of NAC as a ROS (Reactive Oxygen Species) scavenger and a GSH (glutathione) synthesis promoter was reported in our previous investigations and the concentration range was chosen accordingly [52]. Globally, all the compounds tested here are not effective or weakly effective at higher doses as regards metabolic activity reduction, mainly after 48 h. It is therefore plausible to assume that CAI/CO-RM hybrids here tested do not affect cell viability in a non-inflammatory cell system. More in details, after 24 h, percentages of metabolically active cells are found slightly but significantly decreased in a dose-dependent manner in the presence of loading concentrations of compounds **1** (up to 63.73% at 400 μM), **3** (62.88%), **4** (73.33%), **5** (75.22%), **7** (71.40%), **9** (65.69%), and **10** (65.90%). Contrariwise, when mouse macrophages are treated with increasing concentrations of compounds **1**–**10** for 48 h, percentages of metabolic activity are comparable to the one of untreated macrophages set as 100%. Exceptionally, compound **5** significantly increases proliferation of mouse macrophages being percentages of metabolically active cells set at values higher than 100% (Figure S4). In parallel, the antioxidant NAC seems to be not effective in the chosen concentration range, even at the highest dose here administered (Figure S5).

#### 3.5.2. Effect on Cell Viability of Compounds in LPS-Stimulated RAW 264.7 Macrophages after a Short Exposure Time

Macrophages are regarded as critical effectors of inflammation. They express pattern recognition molecules, such as Toll-like Receptor (TLR) 4, to recognize foreign pathogens, remove toxic molecules, and protect against infection. The pro-inflammatory endotoxin or lipopolysaccharide (LPS) is produced by Gram-negative microorganisms and binds to TLR4, triggering the inflammation process [53]. In the present investigation, RAW 264.7 macrophages were stimulated with LPS 0.1 μg/mL to establish pro-inflammatory conditions in vitro. Loading concentrations of LPS up to 25 μg/mL for 48 h were tested on macrophages, disclosing a reduction of cell metabolic activity around 40% already at the lowest dose of 0.1 μg/mL after 24 h of treatment (data not shown). The present cell model was therefore established in accordance with preliminary tests and our previous investigations, where it has been demonstrated that RAW 264.7 macrophages were dramatically sensitive to a 0.1 μg/mL LPS-stimulation in terms of cytokine release and expression of related proteins [39].

**Figure 8:**
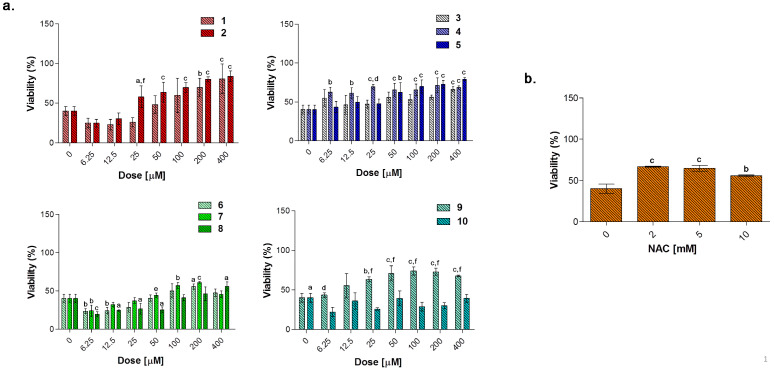
Cell metabolic activity of lipopolysaccharide (LPS)-stimulated (0.1 μg/mL) RAW 264.7 mouse macrophages in the presence of increasing concentrations (0–400 μM) of compounds **1**–**10** (**a**) and NAC (*N*-acetyl cysteine) (0–10 mM) (**b**) after 24 h. Data shown are the means ± S.D. of six replicates and are expressed as percentages of untreated cultures set as 100% (not shown). The 0 μM concentration represents cells stimulated with 0.1 μg/mL alone. (a = *p* < 0.05 between compounds or NAC and cells exposed to LPS alone; b = *p* < 0.01 between compounds or NAC and cells exposed to LPS alone; c = *p* < 0.001 between compounds or NAC and cells exposed to LPS alone; d = *p* < 0.05 between compounds at the same concentration; e = *p* < 0.01 between compounds at the same concentration; f = *p* < 0.001 between compounds at the same concentration).

**Figure 9:**
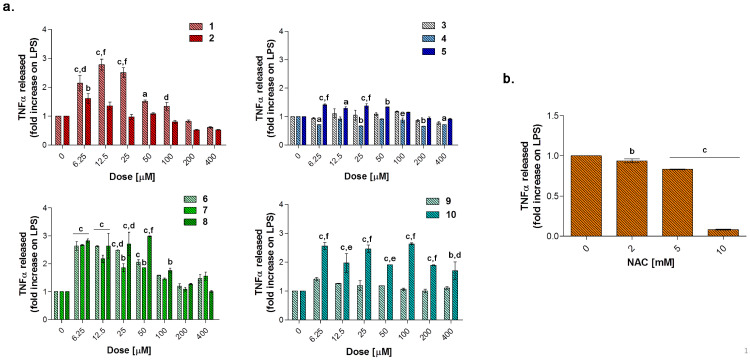
Tumor necrosis factor alpha (TNF-α) released from LPS-stimulated (0.1 μg/mL) RAW 264.7 mouse macrophages in the presence of increasing concentrations (0–400 μM) of compounds **1**–**10** (**a**) and NAC (*N*-acetyl cysteine) (0–10 mM) (**b**) after 24 h. Data shown are the means ± S.D. of six replicates and are expressed as the fold increase of the TNF-α released (pg/mL) from cells stimulated with 0.1 μg/mL LPS alone for 24 h (0 μM) set as 1. (a = *p* < 0.05 between compounds or NAC and cells exposed to LPS alone; b = *p* < 0.01 between compounds or NAC and cells exposed to LPS alone; c = *p* < 0.001 between compounds or NAC and cells exposed to LPS alone; d = *p* < 0.05 between compounds at the same concentration; e = *p* < 0.01 between compounds at the same concentration; f = *p* < 0.001 between compounds at the same concentration).

**Figure 10:**
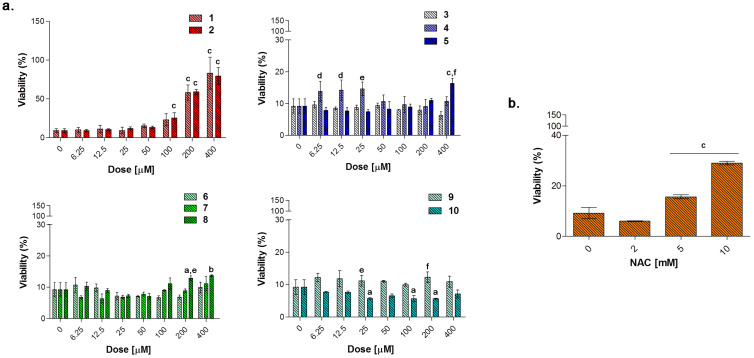
Cell metabolic activity of LPS-stimulated (0.1 μg/mL) RAW 264.7 mouse macrophages in the presence of increasing concentrations (0–400 μM) of compounds **1**–**10** (**a**) and NAC (*N*-acetyl cysteine) (0–10 mM) (**b**) after 48 h. Data shown are the means ± S.D. of six replicates and are expressed as percentages of untreated cultures set as 100% (not shown). The 0 μM concentration represents cells stimulated with 0.1 μg/mL alone. (a = *p* < 0.05 between compounds or NAC and cells exposed to LPS alone; b = *p* < 0.01 between compounds or NAC and cells exposed to LPS alone; c = *p* < 0.001 between compounds or NAC and cells exposed to LPS alone; d = *p* < 0.05 between compounds at the same concentration; e = *p* < 0.01 between compounds at the same concentration; f = *p* < 0.001 between compounds at the same concentration).

In the experimental model here presented, LPS-stimulated cells were afterwards exposed to loading concentrations of compounds **1**–**10** for 24 and 48 h (Figure 8, Figure 9 and Figure 10) to investigate their effect as potential anti-inflammatory molecules and on the TNF-α release. Likewise, NAC was administered for comparison. A 24 h exposure discloses the acesulfame derivatives **1**, **2** and the dihydropyrazole derivative **9** as the best compounds capable of restoring viability in terms of cell metabolic activity in LPS-stimulated cultures (Figure 8a). At length, a significant dose-dependent increase in cell viability can be registered when **1** and **2** are co-administered with LPS 0.1 μg/mL, compared to LPS alone (40.16%), with compound **2** already effective at 25 μM with respect to compound **1** (57.98% and 25.82%, respectively). Over this concentration, the effect of the two compounds is comparable, although cell viability in the presence of **2** is slightly higher than that after compound **1** administration up to 200 and 400 μM (80.08 and 83.97% respectively, for compound **2**). As for the substituted aryl compounds **3**–**5**, they raise cell metabolic activity in a dose-dependent manner, but to a lesser extent compared to **1** and **2**. In details, compound **4** can increase percentages of metabolically active cells starting from the lowest dose of 6.25 μM up to 25 μM (62.66%, 61.15%, and 69.52%, respectively) with respect to LPS alone. After that, percentages become flattened. As for **3** and **5**, the increase of cell viability is strictly dose-dependent starting from 50 μM concentration. The coumarin derivatives **6–8** are slightly effective, with percentages of cell viability not exceeding 60% also at the highest doses. Finally, compounds **9** and **10** show different and opposite effects in the tested concentration range. While the dihydropyrazole derivative (compound **9**) is dose-dependently effective when co-administered with LPS (around 70% of viable cells starting from 50 μM), the benzimidazole derivative (compound **10**) is absolutely not effective. As expected, NAC is capable of counteracting LPS-induced decrease of metabolic activity (Figure 8b). Interestingly, percentages of cell metabolic activity registered are not higher than 64.91% (NAC 5 mM), revealing inhibitors **1** and **2** as more efficient compounds than NAC in counteracting LPS-induced cell viability decrease after 24 h in RAW 264.7 macrophages.

#### 3.5.3. TNF-α Release in LPS-Stimulated RAW 264.7 Macrophages after 24 h

Tumor necrosis factor alpha (TNF-α) signals through two transmembrane receptors, TNFR1 and TNFR2, and regulates several critical cell functions including proliferation, survival, differentiation, and apoptosis. Macrophages are the major producers of TNF-α and are also highly responsive to this cytokine [54]. We have already reported the effect of increasing concentrations of endotoxin in terms of cytokine release in RAW 264.7 macrophages, demonstrating that 0.1 μg/mL LPS considerably enhances TNF-α secretion without reaching saturation of about 400-fold compared to untreated cultures (around 17 pg/mL) [39]. In our experimental model, the value of TNF-α produced by 0.1 μg/mL LPS-stimulated macrophages (2.667 pg/mL, data not shown) is set as 1 and the cytokine release after a co-administration with compounds **1**–**10** is shown as fold increases.

Globally, all the compounds tested here have a similar behavior in terms of TNF-α release after 24 h of exposure (Figure 9a), showing a dose-dependent increase up to a peak and then a fall, resembling a bell curve. The amount of cytokine secreted varies among the different group of derivatives. More in details, the acesulfame-derivative compound **1** shows its TNF-α highest fold increase at 6.25 μM (1.61 folds), while the release of TNF-α increases in a dose-dependent manner up to 12.5 μM for compound **2** (2.78 folds). As for compounds **3**–**5**, only the latter can trigger a slight but significant TNF-α secretion with respect to LPS alone. Among the coumarin-derivatives, compound **7** is the one which displays the TNF-α peak at the lowest dose (2.67 folds at 6.25 μM), followed by compound **6** (2.69 folds at 12.5 μM) and **8** (2.98 folds at 50 μM). Finally, compound **9** shows a flat and not significant TNF-α secretion profile, whereas compound **10** displays an irregular behavior in terms of cytokine released, interchanging peaks (2.51- and 2.45-fold increase at 6.25 μM and 25 μM, respectively) and decreases. As mentioned above, macrophages secrete TNF-α and are also highly responsive to this cytokine and the activation of the cytokine cascade is clinically relevant for the modulation of the inflammatory process. Interestingly, apart from secretion of a repertoire of cytokines/chemokines, macrophages also respond to these products in an autocrine/paracrine manner, thus accentuating the inflammatory response for recruiting other cells involved in the adaptive cell response towards inflammation [54]. A drastic inhibition or a missed activation of the LPS-induced cytokine release as shown for the substituted aryl compounds could be therefore unfavorable. Although induction of pro-inflammatory cytokine expression is critical for a rapid response to tissue trauma or infection, prolonged or deregulated production of these factors may have serious adverse consequences. TNF-α, for example, can be highly cytotoxic, and an inappropriate expression of this cytokine has been linked to a variety of serious pathological conditions [54]. Thus, inflammation is a dynamic and continuous process, with monocytes/macrophages secreting TNF-α as major coordinators. Interestingly, only the acesulfame-derivatives are capable of dramatically decreasing the TNF-α secretion at levels lower than LPS alone. For instance, after 200 and 400 μM compound **1** administration, values of fold increase are assessed at 0.83 and 0.61 compared to LPS alone. In parallel, the TNF-α secretion is even more inhibited in the presence of compound **2** starting from a dose of 100 μM (0.80 folds), being halved with respect to LPS alone at 400 μM (0.52 folds). In the light of a dynamic inflammatory cascade as described above, it is not surprising that low concentrations of dual-acting CAI/CO-RM hybrids **1**, **2**, **6**, **7,** and **8**, increase TNF-α secretion, to counteract inflammation. However, at higher concentrations, these compounds reduce the amounts of TNF-alpha to basal levels and below. As expected, after 24 h of treatment with the antioxidant NAC, a significant dose-dependent decrease of TNF-α secretion can be registered at 5 mM (0.83 folds) and 10 mM (0.07 folds), as we have already reported [42]. It should be noted that, the NAC-induced TNF-α secretion fall is obtained starting from higher doses (millimolar range) with respect to the one registered for the acesulfame-derivatives in the micromolar range. This could be ascribed to their different intracellular molecular targets, being plausible to assume that the acesulfame-derivatives CO-releasing molecules are directly effective on the activation of HO-1, which in turn inhibits the NF-kB-induced cytokine cascade activated by LPS [55]. Contrariwise, although convergent to an increased HO-1 activity through the enhancement of Nrf-2 transcription, the GSH precursor NAC acts on a different pathway which involves several molecular activations prior to activate HO-1 [39].

#### 3.5.4. Biological Effect of Compounds after 48 h

To evaluate the capability of counteracting LPS-induced decrease of cell viability through the TNF-α signaling, RAW 264.7 macrophages were exposed to loading concentrations of compounds **1**–**10** (Figure 10a) and NAC (Figure 10b) for additional 24 h in the presence of LPS 0.1 μg/mL. As expected, NAC is well capable of counteracting the LPS-induced decrease in cell metabolic activity in a dose-dependent manner in the millimolar range. As for compounds tested, the only group capable of restoring cell viability in terms of metabolic activity to percentages comparable with the one of untreated cultures, is represented by the acesulfame-derivatives. More in details, cell viability is dramatically increased starting from compounds **1** and **2** 100 μM (around 25% for both) up to 400 μM (approximately 80% for both) with respect to LPS alone (9.17%). These data can be partly ascribed to the CO-release profile of compounds **1** and **2**, which have been found to deliver CO at low concentrations with step-wised increases over the time with respect to substituted aryl compounds and dihydropyrazole/benzimidazole derivatives (Figure 3). Indeed, as previously mentioned, despite extreme toxicity at high concentrations, an exogenous CO delivery at low concentrations shows a therapeutic potential in the anti-inflammatory process [1]. Furthermore, compounds **6** and **7** release low amounts of CO as the acesulfame derivatives, but they are slightly effective in terms of cell viability restoring, although it is well known that some coumarins have anti-inflammatory effects in LPS-stimulated RAW 264.7 macrophages via the Nrf-2/HO-1 pathway [56].

## 4. Conclusions

Collectively, we described for the first time the synthesis and the biological evaluation of a series of new small molecule hybrids (CAI/CO-RMs). All reported compounds **1**–**10** have been evaluated in vitro both for their selective hCA inhibition and their CO releasing properties studying on the Soret region the Mb-CO absorption spectra. These data have been also corroborated by molecular modelling studies. In addition to their interesting CO releasing properties, five compounds were also demonstrated to act as selective hCA IX and/or XII inhibitors. Successively, we have determined their effectiveness on a murine cell line in terms of metabolic activity and proliferation up to 48 h of treatment. Next, these hybrids were tested on LPS-stimulated cells, mimicking inflammatory conditions in vitro. We therefore observed a counteraction of the inflammatory stimulus at a biological level, being cell metabolic activity restored after 48 h in the presence of compounds **1** and **2** mainly, but also a decreased release of TNF-α. Finally, selected CAI/CO-RMs **1** and **2** revealed better biological and molecular profiles compared to NAC used here as a reference antioxidant compound.

In light of these results, we can consider our new CAI/CO-RM hybrids for the management of human affecting diseases such as inflammation-related ones. They are also important to establish how the organic portion of the molecule, obtained by the hybridization approach, could allow the controlled CO release in order to obtain a pharmaceutically suitable gasotransmitter. We are aware that the structure/release relationships obtained from the in vitro Mb assay do not necessarily reflect the CO release rate in cells. Determination of the CO accumulated in cells, although not always indicative of the expected biological effect exerted by the analyzed DCH compounds [23], will be surely developed as follow up of our studies in the field.

## Figures and Tables

**Table 1:** Inhibition data against hCA I, hCA II, hCA IX, and hCA XII of compounds **1**–**10**, and the standard sulfonamide inhibitor acetazolamide (**AAZ**) by a Stopped-Flow CO_2_ hydrase assay [47].

*K*_I_ (nM) *				
Compound	hCA I	hCA II	hCA IX	hCA XII
**1**	>10,000	>10,000	56.3	788.4
**2**	>10,000	>10,000	>10,000	3462
**3**	>10,000	>10,000	>10,000	>10,000
**4**	>10,000	>10,000	>10,000	>10,000
**5**	>10,000	>10,000	>10,000	>10,000
**6**	>10,000	>10,000	>10,000	4365
**7**	>10,000	>10,000	8112	332.3
**8**	>10,000	>10,000	802.6	540.1
**9**	>10,000	>10,000	>10,000	>10,000
**10**	>10,000	>10,000	>10,000	>10,000
**AAZ**	250	12.1	25.8	5.7

* Mean value from three independent assays (errors were in the range of ±5 to 10%).

## Data Availability

Data are contained within the article.

## Supplementary Materials

The following are available online at https://www.mdpi.com/2076-3921/10/1/56/s1, Figure S1: Chromatograms (HPLC) of compounds **1**–**10**, Figure S2: Correlation between ∆E_calc_ and ∆G_exp_ values, defined for complexes of hCA IX-mimic with **a**, **b**, **1,** and **2**, Figure S3: Correlation between ∆E_calc_ and ∆G_exp_ values, defined for complexes of hCA XII with **a**, **b**, **1,** and **2**, Figure S4: Cell metabolic activity of RAW 264.7 mouse macrophages in the presence of increasing concentrations (0–400 μM) of compounds **1**–**10**. Data shown are the means ± S.D. of six replicates and are expressed as percentages of untreated cultures (0 μM) set as 100% (a = *p* < 0.05 between compounds and untreated cells; b = *p* < 0.01 between compounds and untreated cells; c = *p* < 0.001 between compounds and untreated cells), Figure S5: Cell metabolic activity of RAW 264.7 mouse macrophages in the presence of increasing concentrations (0–10 mM) of NAC. Data shown are the means ± S.D. of six replicates and are expressed as percentages of untreated cultures (0 mM) set as 100% (a = *p* < 0.05 between NAC and untreated cells), Table S1: Inhibition data against hCA I, hCA II, hCA IX, and hCA XII of propargylated precursors and the standard sulfonamide inhibitor acetazolamide (AAZ) by a Stopped-Flow CO_2_ hydrase assay, Table S2: calculated binding energies in solution, ∆E_calc_, and experimental free energies, ∆G_exp_, for hCA IX-mimic and hCA XII, with **1** and **2** and their propargyl precursors, **a** and **b**. R^2^ values are also provided.
