# Supplementary material for: Dual Carbonic Anhydrase IX/XII Inhibitors and Carbon Monoxide Releasing Molecules Modulate LPS-Mediated Inflammation in Mouse Macrophages
Source: Antioxidants (Basel). 2021 Jan 5;10(1):56. doi: 10.3390/antiox10010056 (PMC7824903; doi:10.3390/antiox10010056)
Supplement: Supplementary file 1 [file antioxidants-10-00056-s001.pdf]

antioxidants

MDPI

Supplementary Material

Dual carbonic anhydrase IX/XII inhibitors and Carbon Monoxide Releasing Molecules modulate LPS-mediated inflammation in mouse macrophages

Emanuela Berrino[a], Simone Carradori[b,*], Andrea Angeli[a,c], Fabrizio Carta[a], Claudiu T. Supuran[a], Paolo Guglielmi[d], Cecilia Coletti[b], Roberto Paciotti[b], Helmut Schweikl[e], Francesca Maestrelli[f], Elisabetta Cerbai[a] and Marialucia Gallorini[b,e]

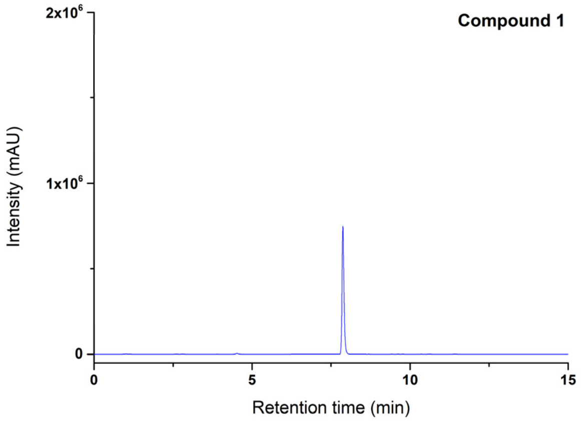

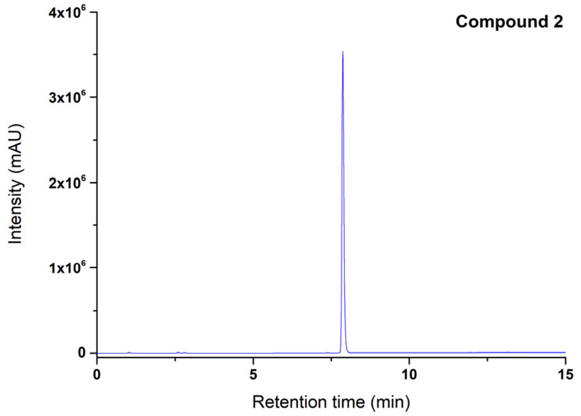

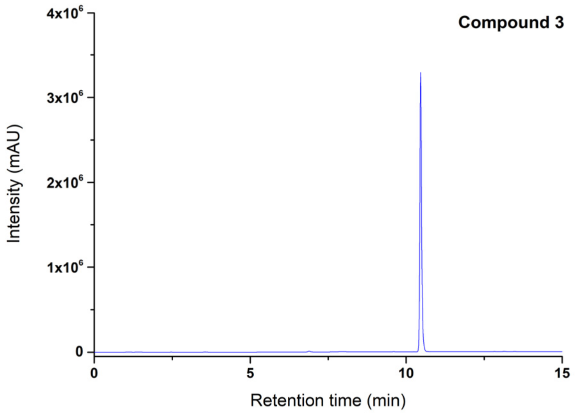
Compound 3
Intensity (mAU)
Retention time (min)
4x10^6
3x10^6
2x10^6
1x10^6
0
0
5
10
15

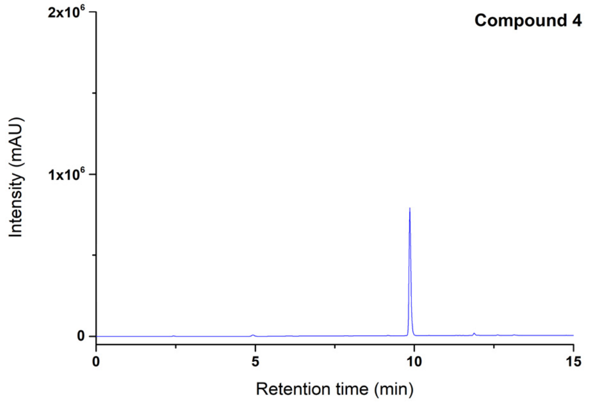
Compound 4
Intensity (mAU)
Retention time (min)
2x10^6
1x10^6
0
0
5
10
15

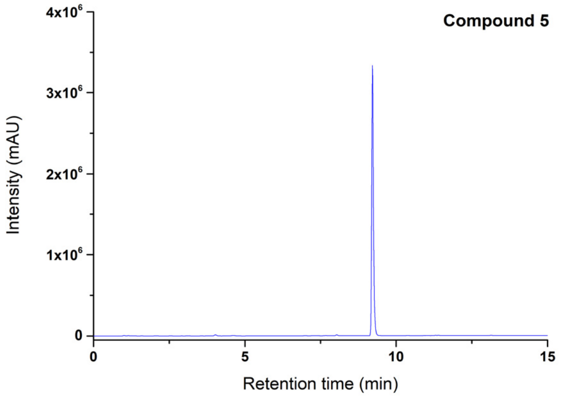
Compound 5
Intensity (mAU)
Retention time (min)
4x10^6
3x10^6
2x10^6
1x10^6
0
0
5
10
15

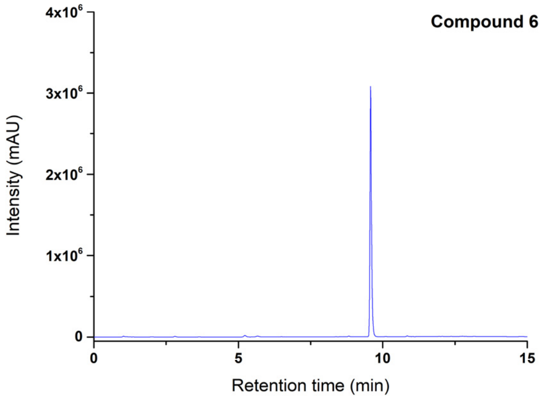
Compound 6
Intensity (mAU)
Retention time (min)

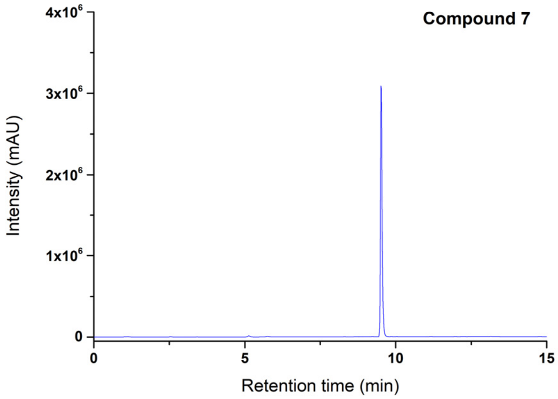
Compound 7
Intensity (mAU)
Retention time (min)

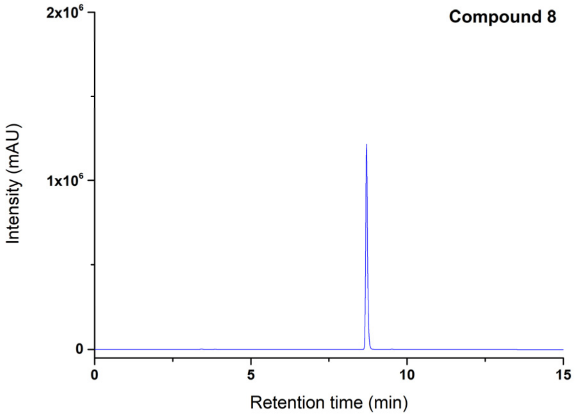
Compound 8
Intensity (mAU)
Retention time (min)

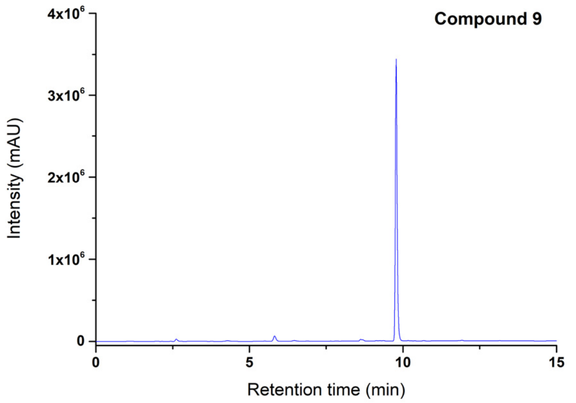

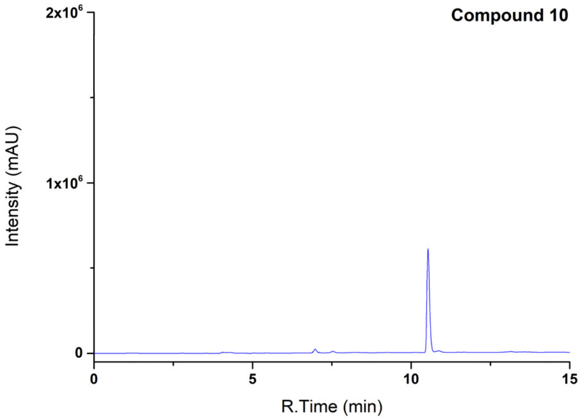

Figure S1. Chromatograms (HPLC) of compounds **1**-**10**.

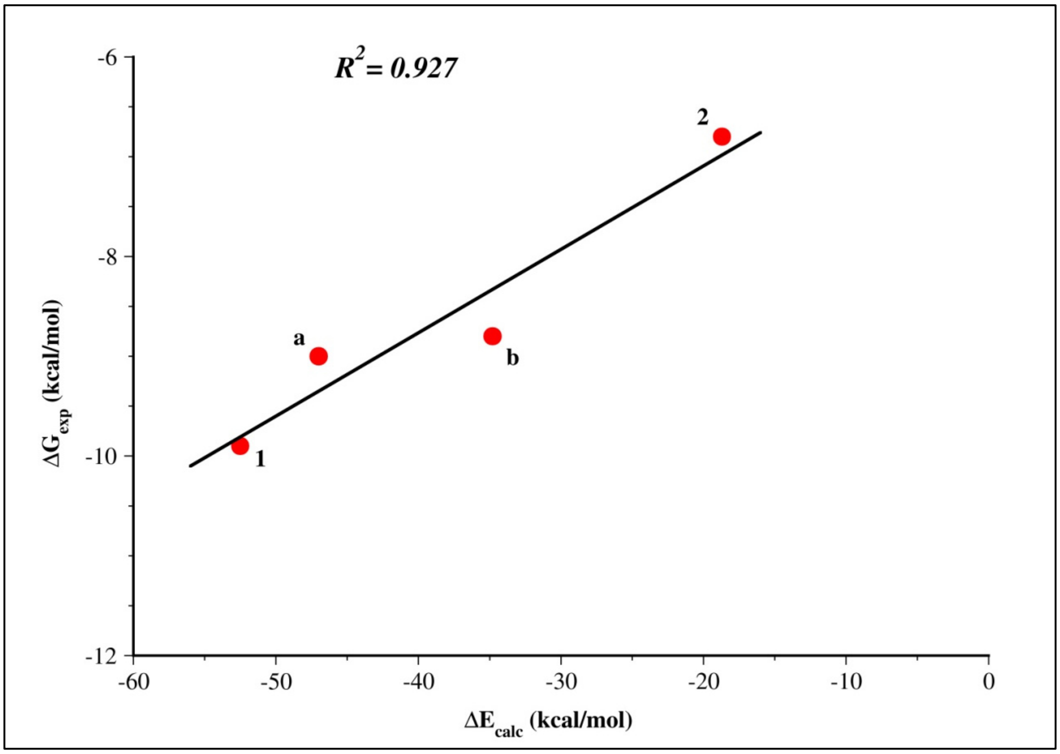

Figure S2. Correlation between ΔE_{calc} and ΔG_{exp} values, defined for complexes of hCA IX-mimic with **a**, **b**, **1** and **2**.

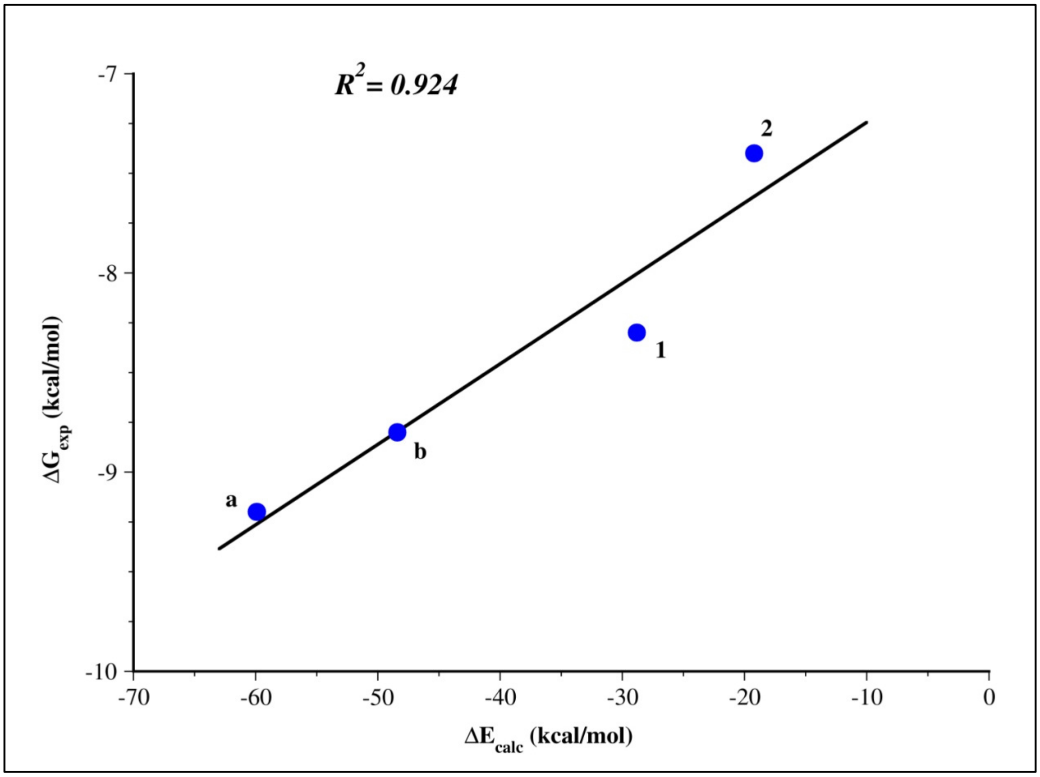

Figure S3. Correlation between ΔE_{calc} and ΔG_{exp} values, defined for complexes of hCA XII with **a**, **b**, **1** and **2**.

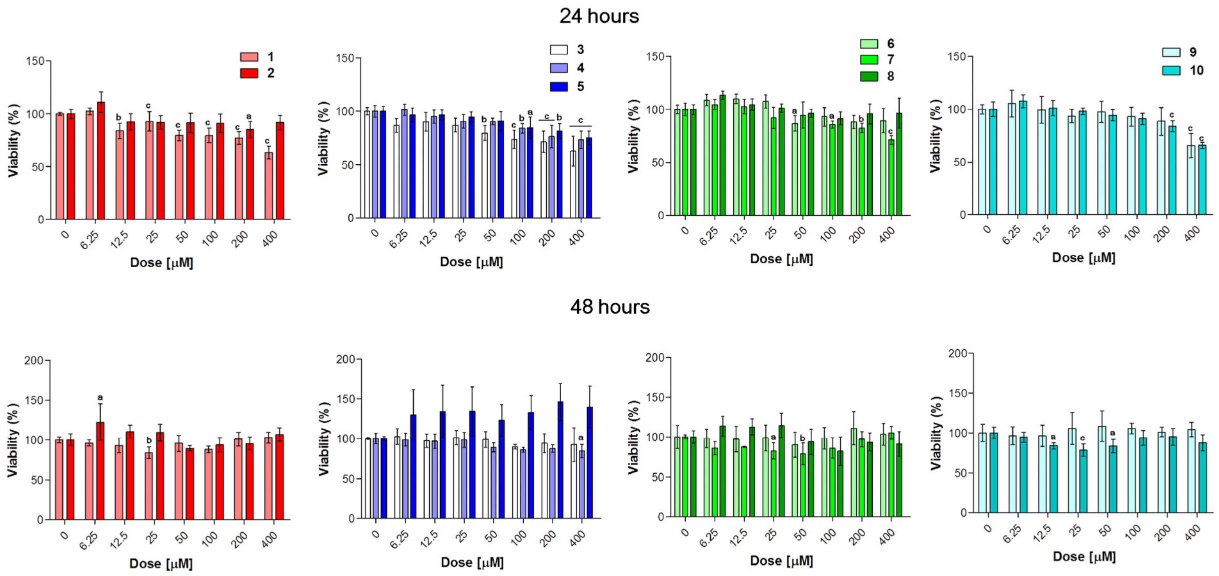

Figure S4. Cell metabolic activity of RAW 264.7 mouse macrophages in the presence of increasing concentrations (0-400 μM) of compounds **1-10**. Data shown are the means ± S.D. of six replicates and are expressed as percentages of untreated cultures (0 μM) set as 100% (a = $p<0.05$ between compounds and untreated cells; b = $p<0.01$ between compounds and untreated cells; c = $p<0.001$ between compounds and untreated cells).

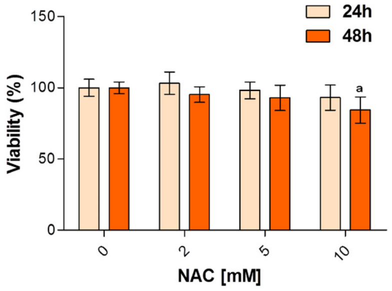

Figure S5. Cell metabolic activity of RAW 264.7 mouse macrophages in the presence of increasing concentrations (0-10 mM) of NAC. Data shown are the means ± S.D. of six replicates and are expressed as percentages of untreated cultures (0 mM) set as 100% (a = $p<0.05$ between NAC and untreated cells).

Table S1. Inhibition data against hCA I, hCA II, hCA IX and hCA XII of propargylated precursors and the standard sulfonamide inhibitor acetazolamide (**AAZ**) by a Stopped-Flow CO_2 hydrase assay.

		K_I (nM)[a]		
**compound**	**hCA I**	**hCA II**	**hCA IX**	**hCA XII**
**a**[b]	>100000	>100000	252.0	178.0
**b**	>100000	>100000	358.0	378.0
**c**	>100000	>100000	>100000	>100000
**d**	>100000	>100000	>100000	>100000
**e**	>100000	>100000	>100000	>100000
**f**	>100000	>100000	695.0	4920
**g**	>100000	>100000	315.5	353.0
**h**	>100000	>100000	>100000	>100000
**i**	>100000	>100000	>100000	>100000
**l**	>100000	>100000	>100000	>100000
**AAZ**	250	12.1	25.8	5.7

[a]Mean value from three independent assays (errors were in the range of ± 5-10%). [b]from ref. (De Monte, C; Carradori, S; Secci, D; D'Ascenzio, M; Vullo, D; Ceruso, M; Supuran, CT. Cyclic tertiary sulfamates: Selective inhibition of the tumor-associated carbonic anhydrases IX and XII by *N*- and *O*-substituted acesulfame derivatives. *Eur. J. Med. Chem.* 2014, *84*, 240-246).

Table S2. calculated binding energies in solution, ΔE_{calc}, and experimental free energies, ΔG_{exp}, for hCA IX-mimic and hCA XII, with **1** and **2** and their propargyl precursors, **a** and **b**. R^2 values are also provided.

**Molecule**	**hCA IX**			**hCA XII**		
	*ΔE_{calc}	*ΔG_{exp}	#K_I	*ΔE_{calc}	*ΔG_{exp}	#K_I
**a**	-47.0	-9.0	252	-59.9	-9.2	178
**1**	-52.5	-9.9	56.3	-28.8	-8.3	788.4
**b**	-34.8	-8.8	358	-48.4	-8.8	378
**2**	-18.7	-6.8	10000	-19.2	-7.4	3462
R^2	0.927			0.924		

*Energy values in kcal/mol; #K_I in nM.
